# *Schistosoma mansoni* Enhances Host Susceptibility to Mucosal but Not Intravenous Challenge by R5 Clade C SHIV

**DOI:** 10.1371/journal.pntd.0001270

**Published:** 2011-08-02

**Authors:** Nagadenahalli B. Siddappa, Girish Hemashettar, Vivekanandan Shanmuganathan, Amma A. Semenya, Elizabeth D. Sweeney, Katherine S. Paul, Sandra J. Lee, W. Evan Secor, Ruth M. Ruprecht

**Affiliations:** 1 Dana-Farber Cancer Institute, Boston, Massachusetts, United States of America; 2 Harvard Medical School, Boston, Massachusetts, United States of America; 3 Centers for Disease Control and Prevention, Atlanta, Georgia, United States of America; 4 Department of Biostatistics and Computational Biology, Dana-Farber Cancer Institute, Boston, Massachusetts, United States of America; Rosetta Genomics, Israel

## Abstract

**Background:**

The high prevalence of HIV-1/AIDS in areas endemic for schistosomiasis and other helminthic infections has led to the hypothesis that parasites increase host susceptibility to immunodeficiency virus infection. We previously showed that rhesus macaques (RM) with active schistosomiasis were significantly more likely to become systemically infected after intrarectal (i.r.) exposure to an R5-tropic clade C simian-human immunodeficiency virus (SHIV-C) than were parasite-free controls. However, we could not address whether this was due to systemic or mucosal effects. If systemic immunoactivation resulted in increased susceptibility to SHIV-C acquisition, a similarly large difference in host susceptibility would be seen after intravenous (i.v.) SHIV-C challenge. Conversely, if increased host susceptibility was due to parasite-induced immunoactivation at the mucosal level, i.v. SHIV-C challenge would not result in significant differences between parasitized and parasite-free monkeys.

**Methods and Findings:**

We enrolled two groups of RM and infected one group with *Schistosoma mansoni*; the other group was left parasite-free. Both groups were challenged i.v. with decreasing doses of SHIV-C. No statistically significant differences in 50% animal infectious doses (AID_50_) or peak viremia were seen between the two groups. These data strongly contrast the earlier i.r. SHIV-C challenge (using the same virus stock) in the presence/absence of parasites, where we noted a 17-fold difference in AID_50_ and one log higher peak viremia in parasitized monkeys (*P*<0.001 for both). The lack of significant differences after the i.v. challenge implies that the increased host susceptibility is predominantly due to parasite-mediated mucosal upregulation of virus replication and spread, rather than systemic effects.

**Conclusions:**

The major impact of schistosome-induced increased host susceptibility is at the mucosal level. Given that >90% of all new HIV-1 infections worldwide are acquired through mucosal contact, parasitic infections that inflame mucosae may play an important role in the spread of HIV-1.

## Author Summary

Parasitic infections have been postulated to increase host susceptibility to HIV-1. We previously demonstrated that rhesus monkeys with active schistosomiasis were significantly more likely to become systemically infected after intrarectal exposure to an R5-tropic clade C simian-human immunodeficiency virus then were parasite-free control animals. However, we could not address whether parasites exert their effect at the mucosal level or systemically. To address the latter possibility, we measured the virus doses needed to achieve systemic infection after intravenous exposure of parasite-free or parasite-positive monkeys using the identical virus stock. None of the viral parameters tested in these two groups of monkeys were statistically significantly different. These results suggest that schistosomiasis modulates susceptibility to immunodeficiency virus acquisition predominantly at the mucosal level. Treatment for parasitic infections in populations at higher risk for HIV-1 acquisition could represent a cost-effective approach to slow the spread of HIV-1, which is predominantly transmitted through mucosal routes.

## Introduction

More than 20 million people in sub-Saharan Africa are infected with HIV-1 (www.UNAIDS.org) [1]. As in the case of HIV/AIDS, a disproportionate number of the estimated 207 million people in the world who have schistosomiasis live in Africa. Because of the high prevalence of schistosomiasis and soil-transmitted helminth infections in this region and the apparent disparity of HIV-1 transmission rates compared to more industrialized countries, parasitic worm infections have been hypothesized to contribute to the transmission and progression of immunodeficiency virus infections [2], [3], [4], [5]. This hypothesis has been supported by studies showing increased expression of HIV-1 co-receptors and elevated viral loads in individuals with active helminth infections compared to persons who have been successfully treated for their parasites [6], [7], [8]. However, studies in humans have so far been unable to definitely answer, or even directly address, whether schistosomiasis and soil-transmitted helminth infections increase an individual's susceptibility to acquiring HIV-1.

To investigate the above hypothesis, a biologically relevant nonhuman primate model would be useful. We previously constructed and characterized a clade C R5 simian-human immunodeficiency virus (SHIV-C), termed SHIV-1157ipd3N4 [9]. This virus encodes an envelope gene of the HIV-1 clade that predominates in regions of the world where helminth infections are highly prevalent. SHIV-1157ipd3N4 exclusively uses CCR5 for cell entry and thus reflects the tropism of virtually all mucosally transmitted strains of HIV-1 (>90% of all new HIV-1 acquisitions occur mucosally). Like HIV-1 infection in humans, SHIV-1157ipd3N4 infection in rhesus macaques (RM) progresses slowly to late disease stages [10], [11], [12]. We also showed that SHIV-1157ipd3N4 was transmissible across intact rectal, vaginal and oral mucosae and that the relative mucosal transmissibility followed the risk order of sexual HIV-1 acquisition among humans [12]. Therefore, SHIV-1157ipd3N4 reflects the salient biologic features of HIV-1 transmission among humans.

Using this R5 SHIV-C model, we tested the effect of *Schistosoma mansoni* infection using parasite-free and parasitized RM exposed intrarectally (i.r.) to SHIV-C [13]. We found that the virus dose needed to achieve viremia in 50% of the animals by the i.r. route (i.e., the 50% animal infectious dose (AID_50_-i.r.)) was 17-fold lower in RM with acute schistosomiasis compared to parasite-free animals. However, we could not differentiate whether the observed effects of schistosome infection on SHIV-C acquisition were primarily the result of mucosal effects, such as inflammation and local reactions caused by the passage of parasite eggs, or were due to systemic effects.

To address the latter possibility, we intravenously (i.v.) challenged schistosome-free and schistosome-infected RM with the identical stock of SHIV-1157ipd3N4 as that used in the i.r. study [13]. We followed a similar experimental design to determine the minimal infectious virus dose and the AID_50_ for the i.v. route (AID_50_-i.v.) by endpoint titration. The AID_50_-i.v differed only 3.3 fold between parasite-free and *S. mansoni-*positive RM, a difference that was not statistically significant. The magnitude of this difference was much smaller than that observed in the i.r. SHIV-C exposure study, suggesting that parasitic infections modulate host susceptibility to lentivirus acquisition predominantly at mucosal levels.

## Materials and Methods

### Virus

SHIV-1157ipd3N4 [9], a pathogenic R5-tropic SHIV-C infectious molecular clone, is a monkey-adapted, late form of SHIV-1157i, and encodes most of the *env* sequences of a primary HIV-1 clade C strain isolated from a recently infected Zambian infant. SHIV-1157ipd3N4 was engineered with an additional NF-kB site per long terminal repeat (LTR) in order to enhance viral replication. The methodology used to construct this virus and its biology are described elsewhere [9], [10], [11], [12]. Both the early and late forms of our SHIV-C were pathogenic in RM, although disease progression was somewhat slow, with AIDS developing approximately 2.5 - 5.5 years post-inoculation [10], [12].

### Animals

Chinese-origin adult female RM were housed at the animal facility of the Centers for Disease Control and Prevention (CDC) in Atlanta, GA. This study was carried out in strict accordance with the recommendations in the Guide for the Care and Use of Laboratory Animals of the U.S. Public Health Services/National Institutes of Health, as well as according to the recommendations in the Weatherall report on “The Use of Non-human Primates in Research” (http://www.acmedsci.ac.uk/images/project/nhpdownl.pdf). CDC facilities are fully accredited by the Association for Assessment and Accreditation of Laboratory Animal Care International. Animal experiments were approved by the Institutional Animal Care and Use Committees at the CDC (IACUC ID: 1522) and the Dana-Farber Cancer Institute via a Collaborating Institution Animal Use Agreement. Because the experiments described here involved a virus that may cause an incurable disease, such as AIDS, discomfort, stress and pain may occur. Animals were closely monitored and observed for development of disease at least twice daily. If the animals are determined to be under stress or in discomfort, appropriate anesthetics and/or analgesics are administered as directed by the clinical veterinary staff. Euthanasia is also an option should treatment not alleviate stress. In the current study, no untoward clinical problems were noted, and none of the virus-infected monkeys progressed to AIDS. Animals were free of *S. mansoni* infection at the time the study was initiated.

### *S. mansoni* inoculation

Animals were anesthetized with ketamine and exposed percutaneously with 500 cercariae of a Puerto Rican strain of *S. mansoni*. An area on the abdomen was shaved, and cercariae were placed within a metal ring on the skin for 30 min to allow penetration. To monitor infection, fresh stool was obtained and processed by formalin-ethyl acetate sedimentation and concentration. Schistosome eggs were counted microscopically. White blood cell counts (WBC) and percent eosinophils were calculated using standard methods. Hematology results, as well as CD4 and CD8 T-cell counts and ratios were determined by the Pathology Laboratory of the Yerkes National Primate Research Center (Atlanta, GA). There was no evidence of fever, diarrhea, weight loss, or dysentery in these animals.

### I.v. inoculation of virus

Anesthetized macaques were inoculated i.v. with 1 ml total volume of various dilutions of an identical SHIV-1157ipd3N4 stock that had been grown in RM PBMC. All animals were exposed within 1 hr after the viral stock was thawed.

### Quantitation of viral RNA loads and simian cytokine mRNAs

Peripheral blood samples were obtained by venipuncture and collected into Vacutainer cell-preparation tubes containing sodium citrate (Becton Dickinson, Rutherford, NJ). Immediately after collection, plasma and PBMC were separated and quick-frozen. Plasma samples were stored at −80°C until viral RNA loads were assessed by real-time RT-PCR [14], [15]. A quantitative real-time RT-PCR assay based on TaqMan chemistry was utilized to measure total mRNA levels for the cytokines IL-2, IL-4 and IL-10 using previously described primers and protocols [28]. mRNA expression was normalized using primers unique for the housekeeping gene, GAPDH.

### Statistical analyses

Calculation of AID_50_ values and statistical comparison of infectious doses were performed using the method of Spouge [16]. Peak viral RNA loads were compared using the Wilcoxon rank-sum test. All the reported *P*-values are based on two-sided testing.

## Results

### Determination of SHIV-C doses required for systemic infection after i.v. challenge of naïve RM

To establish the minimum dose of virus required to systemically infect non-parasitized control animals, seven macaques without schistosome infection were inoculated iteratively with different doses of virus via the i.v. route and monitored for systemic SHIV-C infection. If an animal became successfully viremic after the first dose of virus, the next animal was inoculated with a lower dose. Plasma viral RNA loads were monitored using a previously published sensitive RT-PCR method [14], [15] and animals with viral loads >10,000 copies/ml were considered to be systemically infected. Using this procedure and statistical analysis according to the method of Spouge [16], we determined that the AID_50_-i.v. in control animals was 1 ml of a 1∶151,000 dilution of the viral stock (Table 1).

**Table 1:** Intravenous titration of SHIV-1157ipd3N4.

Animal	Animalname	Virus dilution	Systemic infection	Peak viral RNA(copies/mlx10^6^)	AID_50_	95% CI
Parasite-free	51850	1∶5,000	+	6.2		
(n = 7)	61891	1∶25,000^a^	+	7.6		
	61535	1∶25,000^a^	+	2.6		
	61542	1∶50,000	+	8.4^b^		
	DP54	1∶100,000	+	21.2^b^		
	DR13	1∶100,000	+	4.8^b^		
	60980	1∶150,000	+	4.7		
	61891	1∶250,000	–	–		
	61535	1∶500,000	–	–		
				Median 6.2	1∶151,000	1∶51,900 to 1∶442,000
*S. mansoni^+^*	51948	1∶100,000	+	17.6		
(n = 8)	DN3P	1∶100,000^c^	+	10.1		
	61855	1∶100,000^c^	+	7.8		
	61707	1∶250,000	+	5.4^b^		
	DP4D	1∶250,000^c^	+	5.9		
	61951	1∶500,000	+	19.3		
	00216	1∶500,000	–	–		
	RQ4652	1∶750,000	–	–		
	DN3P	1∶1,000,000	–	–		
	61855	1∶1,500,000	–	–		
	DP4D	1∶5,000,000	–	–		
				Median 9.0	1∶521,000	1∶203,000 to 1∶1,340,000(*P* = 0.3 parasite-free vs. *S. mansoni* ^+^)

a These animals were first exposed to a 1∶250,000 and 1∶500,000 dilution of the virus. When infection failed to occur after the initial dose, the monkeys were re-exposed to a 1∶25,000 dilution, which proved to be infective.

b Infection in these animals peaked at week 3.

c These animals were first exposed to 1∶1,000,000, 1∶1,500,000 and 1∶5,000,000 dilution of the virus. After infection failed to occur, the monkeys were re-exposed to 1∶100,000 and 1∶250,000 dilution of virus, which was infective.

Although we initially enrolled 8 RM into the two groups, 1 RM was lost during quarantine due to unrelated causes. The prior i.r. study challenge study also used 8 RM per group [11].

### Determination of SHIV-C doses required for systemic infection after i.v. challenge of parasitized RM

**Figure 1:**
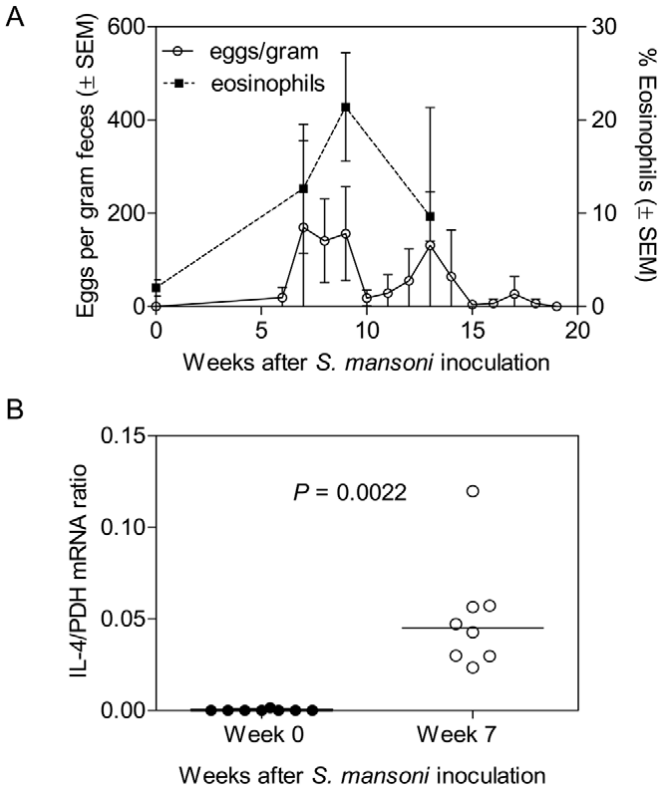
Infection of Chinese-origin RM with *S. mansoni* and IL-4 mRNA expression levels. (A) Egg counts (per gram feces) in stool samples collected from the *S. mansoni*-infected animals. Data represent the average of three consecutive values ± SEM. Percentage of eosinophils was plotted as a function of time (± SEM). (B) Ratios of IL-4 mRNA expression relative to the expression of the housekeeping gene (GAPDH) in PBMC before exposure to *S. mansoni* (week 0) and at week 7 (before exposure to SHIV-C).

First, eight RM were exposed percutaneously to 500 cercariae, the infectious life cycle stage of schistosomes. Successful parasite infection was confirmed by the detection of eggs in the stool beginning at 6 weeks post-exposure (Fig. 1A). There was a concomitant increase in eosinophils (Fig. 1A). The magnitude and time course of infection were similar to our previous studies of macaques infected with the same number of cercariae [13], [17]. Next, peripheral blood mononuclear cells (PBMC) of parasite-infected and control animals were isolated and analyzed for expression of cytokines IL-2, IL-4 and IL-10. Cytokine mRNA levels were normalized against those of the house keeping gene glyceraldehyde 3-phosphate dehydrogenase (GAPDH). The difference in IL-4 mRNA levels before and after schistosome infection was statistically significant (*P*  =  0.0022) (Fig. 1B). This result confirmed the previously noted shift to a T-helper type 2 (Th2) response that is typical in acute *S. mansoni* infections [13], [17].

**Figure 2:**
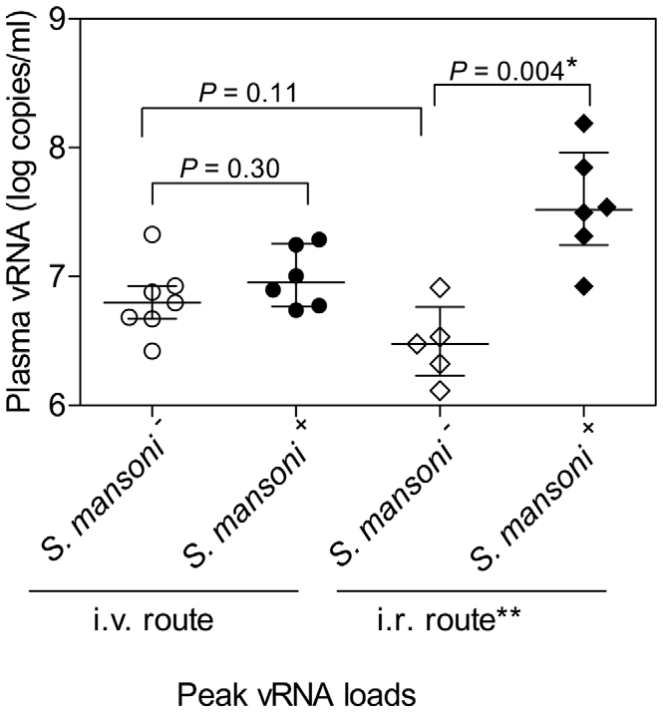
Peak viral RNA loads. Peak viral RNA loads were compared between parasite-free and *S. mansoni*-infected RM in i.v and i.r. challenge experiments. *, *P*  =  0.004 was statistically significant after the Bonferroni correction (since it was below 0.017). **, previously published data were used for comparison [13]; those studies had been performed with the identical stock of SHIV-1157ipd3N4 used here.

**Figure 3:**
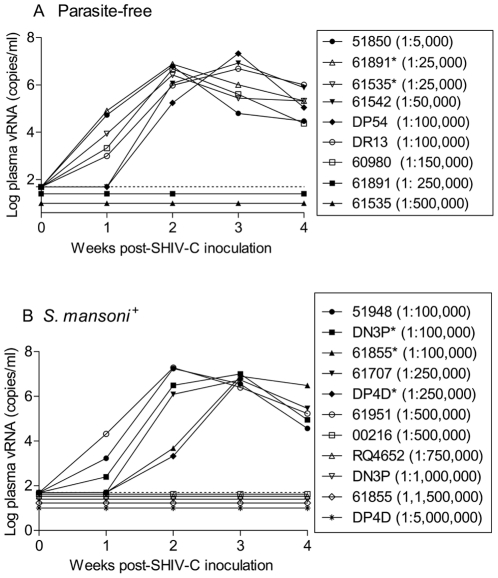
Viral RNA loads of parasite-free and *S. mansoni*-infected RM after SHIV-C challenge. (A & B) viral RNA loads were compared between parasite-free and *S. mansoni*-infected RM during acute infection. During the first four weeks post-challenge, plasma samples were analyzed weekly for viral RNA loads. The horizontal dotted line indicates the lower limit of detection in the PCR assay (<50 viral RNA copies/ml) [14], [15]. *, animals were first exposed to higher dilution of the virus as indicated in the figure. If systemic infection failed to occur after the initial dose, the monkeys were re-exposed to a lower virus dilution, which proved to be infective.

After establishing active schistosomiasis, we repeated the same iterative procedure of i.v. SHIV-C challenges described above. The minimal infectious dose to establish systemic SHIV-C infection in schistosome-infected animals (1∶500,000) was 3.3 fold lower than that in non-parasitized controls (1∶150,000) (Table 1) (Fig. 2), but this difference was not statistically significant. Next, we used the method of Spouge [16] and determined that the AID_50_-i.v. in parasitized RM was 1 ml of a 1∶521,000 dilution of the viral stock compared to 1 ml of a 1∶151,000 dilution in parasite-free RM. This difference was also not statistically significant. Mean peak viral RNA loads in parasitized RM with systemic SHIV-C infection after i.v. challenge were not statistically different from those in RM without schistosomiasis (Fig. 2, 3). In essence, none of the viral parameters showed any statically significant differences between parasite-free and *S. mansoni*-positive RM after i.v. SHIV-C challenge. This is in stark contrast to RM challenged by the i.r. route (Fig. 2) [13]. Of note, peak viral RNA loads observed in parasite-free RM were similar after i.v. and i.r. SHIV-C challenges (Fig. 2).

### Comparison of viral parameters between RM challenged by the i.r. or i.v. routes in the presence or absence of parasites

The composite data for the i.r. and i.v. challenges using the same stock of SHIV-1157ipd3N4 in control and *S. mansoni*-infected RM are shown in Table 2. As previously reported in the i.r. challenge study [13], the AID_50_-i.r. was 17-fold lower in monkeys with acute schistosomiasis compared to control RM. This difference was highly significant (*P*<0.001) [13]. In contrast, in the current i.v. challenge study, this difference was only 3.3 fold and was not statistically significant. These data imply that the increased host susceptibility to SHIV-C associated with schistosome infection is predominantly due to immunoactivation at the mucosal level and not due to systemic effects.

**Table 2:** Relative infectivity of different SHIV-C challenge routes in Chinese-origin rhesus monkeys.

Parasiteinfection	Route ofexposure	Highest virus dilution yielding systemic infection*	AID_50_ (μl)	Relative susceptibility to systemic infection(normalized)
None	intrarectal	1∶50	24.6	1
None	intravenous	1∶150,000	0.0034	7,235
*S. mansoni*	intrarectal	1∶300	1.4	17.6
*S. mansoni*	intravenous	1∶500,000	0.0019	737

*: defined by plasma viral RNA load >10^4^ copies/ml.

Our data also allow a direct comparison of the virus doses needed to achieve systemic infection after i.v. versus i.r. SHIV-C challenge. In parasite-free RM, the AID_50_-i.r. was extrapolated to be 24.6 µl of the undiluted virus stock. In contrast, the AID_50_-i.v. was a mere 0.0034 µl (3.4 nl). We reasoned that these AID_50_ values are indirectly proportional to the relative risks of virus acquisition via a given route of exposure. Thus, we converted the AID_50_ values to 1/AID_50_ and normalized the value for the i.r. route to 1. The ratio of 1∶7,235 reflects the much greater susceptibility of parasite-free hosts to i.v. challenge compared to i.r. exposure. Interestingly, this ratio was much lower for *S. mansoni*-infected RM (7.6∶737 or 1∶42). This additional measure also reflects that *S. mansoni*-induced mucosal alterations preferentially increase host susceptibility to SHIV-C.

## Discussion

Here we showed the results of end-point titrations of an identical SHIV-C stock as a function of the challenge route (i.v. versus i.r.) and the presence or absence of parasites in Chinese-origin RM. Together, our data showed: 1) the difference in AID_50_-i.v. for parasite-positive vs. parasite-free RM was 3.3-fold, which was not statistically significant; 2) in contrast, the difference in AID_50_-i.r. for parasite-positive vs. parasite-free RM was 17-fold (*P*<0.001) with very similar numbers of RM per experimental group; and 3) peak viral RNA loads differed only among i.r. challenged but not i.v. challenged groups of schistosome positive vs. parasite-free RM.

The fact that the 3.3-fold difference in AID_50_-i.v. was not statistically significant, whereas the difference for the AID_50_-i.r. was, needs to be interpreted with caution given the constraints of our study. Primate studies with their inherently high costs limit the numbers of experimental animals that can be enrolled. Much larger group sizes than we were able to use may find that parasitized hosts challenged intravenously develop systemic infection at statistically significantly lower viral inocula compared to parasite-free hosts. Our current data would predict that such a difference would be smaller than that for the mucosal virus challenge.

Overall, our data indicate that the increased host susceptibility to the R5 SHIV-C is predominantly due to parasite-induced changes at the mucosal level. We postulate that host factors of the mucosal innate and adaptive immune systems will be implicated. The nature of our virus challenge study mandated not disturbing the mucosa for tissue sampling. Future studies should seek to identify the exact mechanism(s) involved in mucosally-mediated upregulation of host susceptibility to an R5 AIDS virus in the presence of acute *S. mansoni* infection.

The increased susceptibility of *S. mansoni*-infected monkeys to i.r. viral exposure may be related to the passage of eggs from the bloodstream, where the adult worms reside, to the lumen of the gut. Egg excretion in schistosome-infected hosts is dependent on cellular immune responses [18], [19] and is thought to be facilitated by the granulomatous response to parasite eggs [18]. As a result, schistosomiasis is associated with increased numbers of activated T cells in the intestinal mucosa, along with disruption of the epithelial cell layer integrity as eggs exit the tissue. However, this does not necessarily result in bacterial translocation and transmission. Although a recent study in humans demonstrated an overall increased level of lipopolysaccharides (LPS) in the plasma of parasite-infected Kenyan individuals compared to uninfected controls, no differences were seen when LPS levels were compared between individuals with active egg excretion compared to those without [20]. Similarly, we found no evidence of elevated LPS levels in the plasma of schistosome-infected RM in a previous study (unpublished data).

In *S. haematobium* infections, adult worms live in the blood vessels surrounding the bladder instead of the intestine. Eggs are excreted into the bladder and pass out of the host in the urine. However, the worms can reside in other areas of the urogenital vasculature and eggs often become lodged in other nearby organs, especially in women, and can cause female urogenital schistosomiasis (FUS). The latter is associated with “sandy patches” (yellow, grainy areas), neovascularization, and contact bleeding of the cervix and vaginal wall as a result of the inflammation in response to the eggs. In studies in Zimbabwe and Tanzania, FUS was associated with a 2.7 to 4-fold higher risk of HIV-1 acquisition [4], [21]. Furthermore, the scarring of the cervix and vaginal wall caused by FUS may increase a woman's risk for HIV-1 infection throughout life if she does not receive treatment at a young age [22], [23]. This has led to suggestions that treatment of schistosomiasis could be a low-cost intervention to reduce transmission of HIV-1 in areas of sub-Saharan Africa where this parasite is endemic.

While our primate model studies have focused on the role of parasites in facilitating lentiviral acquisition, other investigators have examined the role of parasites on HIV-1 disease progression. Parasitic worms elevate viral loads and exacerbate destruction of CD4 cells in chronic AIDS virus infection. Both studies in experimental animals [17], [24], [25] and intent-to-treat studies in people with either schistosomiasis [6] or soil-transmitted helminthes [8] showed elevated viral replication in groups with active parasitic infections compared to control groups that were never infected with schistosomes or that had received treatment for their worms.

Helminth infections in pregnant women, with associated higher viral loads, may also increase the risk of mothers transmitting HIV-1 infections to their infants [26]. The majority of maternal HIV-1 transmissions are thought to occur intrapartum via mucosal exposure of the fetus [27]. It is possible that parasite infection of the mother increases the HIV-1 burden in cervico-vaginal secretions, thereby increasing the risk of passing the virus to the infant.

Our current study, together with previous reports, clearly suggests that schistosomiasis increases the risk of immunodeficiency virus acquisition predominantly at the mucosal level. While the conclusions of our earlier intrarectal challenge study are not directly applicable to other routes of HIV-1 exposure among humans, our primate model data provide a proof of concept that alterations in the mucosal environment enhance HIV-1 transmission in schistosome-infected hosts. Our conclusions are also consistent with studies showing FUS as a risk factor for HIV-1 infection and the greater role of heterosexual transmission of HIV-1 in sub-Saharan Africa than in more industrialized countries that do not have endemic schistosomiasis. Thus, treatment for parasitic infections in populations at high risk for HIV-1 acquisition could represent a cost-effective approach to slow the spread of HIV-1. Furthermore, treatment of parasites in individuals with HIV-1 coinfection could lower viral RNA loads, which in turn would decrease the infectivity of such persons and slow their HIV-1 disease progression.
